# Exosomes Derived From Human Gingival Mesenchymal Stem Cells Attenuate the Inflammatory Response in Periodontal Ligament Stem Cells

**DOI:** 10.3389/fchem.2022.863364

**Published:** 2022-04-06

**Authors:** Jiayao Sun, Zhiguo Wang, Peng Liu, Yingzhe Hu, Tingting Li, Jianbo Yang, Pengyu Gao, Quanchen Xu

**Affiliations:** ^1^ Department of Stomatology, The Affiliated Hospital of Qingdao University, Qingdao, China; ^2^ School of Stomatology of Qingdao University, Qingdao, China; ^3^ Department of Burn and Plastic Surgery, The Affiliated Hospital of Qingdao University, Qingdao, China; ^4^ Department of Surgery, Qingdao West Coast New Area People’s Hospital, Qingdao, China; ^5^ Department of Stomatology, Weihai Stomatological Hospital, Weihai, China

**Keywords:** exosomes, periodontal ligament stem cells, gingival mesenchymal stem cells, NF-κB, Wnt5a, inflammatory microenvironment, periodontitis

## Abstract

This study aimed to explore the effects of exosomes derived from human gingival mesenchymal stem cells (GMSC-Exo) on the inflammatory response of periodontal ligament stem cells (PDLSCs) in an inflammatory microenvironment in order to restore the regenerative potential of PDLSCs, which promotes periodontal tissue regeneration in patients with periodontitis. Periodontitis is a chronic infectious disease characterized by periodontal tissue inflammation and alveolar bone destruction. PDLSCs are regarded as promising seed cells for restoring periodontal tissue defects because of their ability to regenerate cementum/PDL-like tissue and alveolar bone. However, PDLSCs in the inflammatory environment show significantly attenuated regenerative potential. GMSC-Exo have been reported to have anti-inflammatory and immunosuppressive properties. In this study, we investigated the effects of GMSC-Exo on the inflammatory response of PDLSCs induced by lipopolysaccharides (LPS). LPS was used to simulate the inflammatory microenvironment of periodontitis *in vitro*. GMSC-Exo were extracted from the culture supernatant of GMSCs by ultracentrifugation. We found that GMSC-Exo attenuated the inflammatory response of PDLSCs induced by LPS. Furthermore, compared to treatment with LPS, treatment with GMSC-Exo attenuated the expression of NF-κB signaling and Wnt5a in LPS-induced PDLSCs. In conclusion, we confirmed that GMSC-Exo could suppress the inflammatory response of PDLSCs by regulating the expression of NF-κB signaling and Wnt5a, which paves the way for the establishment of a therapeutic approach for periodontitis.

## Introduction

Periodontitis is a chronic complex inflammatory disease with several etiologic and causative factors, principally infection with pathogenic microflora (Meyle and Chapple, 2015). Lipopolysaccharides (LPS) from the microflora are considered the major factor in the pathological events. LPS lead to the dysregulation of host immuno-inflammatory response and the release of a large number of inflammatory factors, such as TNF-α and IL-1β, which eventually result in periodontitis (Bickel et al., 2001). Periodontitis is characterized by chronic inflammation of periodontal support tissue, leading to tooth loosening and displacement. Epidemiologic research has shown that periodontitis is associated with a variety of systemic diseases (Bartold, 2012; Cullinan and Seymour, 2013). Currently, severe periodontitis has become a global burden (Barbero et al., 2019), which makes it crucial to eliminate periodontal inflammation and promote periodontial regeneration. One strategy is utilizing the regenerative potential of human periodontal ligament stem cells (PDLSCs) isolated from periodontal ligaments (PDLs), which can generate cementum/PDL-like tissue (Seo et al., 2004; Trubiani et al., 2019). PDLSCs in the inflammatory environment show significantly attenuated osteogenic differentiation potential, hindering the regeneration of periodontal tissues. Therefore, it is necessary to diminish the inflammatory response and restore the therapeutic potential of PDLSCs.

Human gingival mesenchymal stem cells (GMSCs) derived from the human gingiva can be isolated with minimally invasive techniques. Emerging evidence shows that GMSCs have excellent anti-inflammatory and immunomodulatory effects (Zhang et al., 2009; Li, Zhao, and Ge, 2016; Zhou et al., 2020). Previous work has shown that GMSCs can promote alveolar bone regeneration in a mouse model of periodontitis accompanied by hyperlipidemia and reduce the inflammatory response by inhibiting the activation of M1 macrophages (Hong et al., 2019). There are a variety of studies supporting the idea that mesenchymal stem cells (MSCs) play a therapeutic role in a paracrine manner (Fu et al., 2019). Exosomes (Exo), an important paracrine product of MSCs, have a therapeutic potential similar to that of their source cells, which has attracted increasing attention (Levy et al., 2020). As a novel cell-free therapy, exosomes derived from MSCs (MSC-Exo) not only have immuno-inflammatory and modulatory capabilities similar to those of MSCs but also overcome numerous disadvantages of cellular therapy (Jafarinia et al., 2020). Previous studies have found that exosomes derived from GMSCs (GMSC-Exo) can inhibit the activation of M1 macrophages and attenuate the inflammatory response (Wang et al., 2020; Zhang W et al., 2021). It has been demonstrated that GMSC-Exo play an important role in chondrogenesis (Mao et al., 2018), wound healing (Shi et al., 2017; Kou et al., 2018), preventing periodontal bone loss (Nakao et al., 2021), promoting rat sciatic nerve regeneration (Rao et al., 2019), and taste bud regeneration (Zhang Y et al., 2019). Nevertheless, the effect of GMSC-Exo on PDLSCs in the inflammatory microenvironment is unknown.

Accumulating evidence suggests that the nuclear factor kappa-light-chain enhancer of activated B cells (NF-κB) signaling pathway and Wnt family member 5a (Wnt5a) are significant for the development and disease of periodontal tissues (Kurgan and Kantarci, 2018; Wei et al., 2021). It has been reported that inhibition of the NF-κB signaling pathway downregulated the overexpression of Wnt5a induced by injury (Sucre et al., 2020). In addition, LPS-induced activation of Wnt5a can indirectly regulate NF-κB expression through JNK signaling (Yu et al., 2020). Zhao et al. (2015) proposed that the upregulation of Wnt5a mediated a positive feedback loop in the inflammatory response through the PI3K/AKT/NF-κB signaling pathway. Therefore, we investigated whether the inhibitory effect of GMSC-Exo on the inflammatory response of PDLSCs was related to both the NF-κB signaling pathway and Wnt5a.

Based on the aforementioned findings, after treatment with LPS (10 μg/ml), a similar inflammatory microenvironment was established. We speculate that GMSC-Exo might play an important role in regulating the inflammation of PDLSCs that then might contribute to facilitating periodontal tissue regeneration, and GMSC-Exo might exert its effects by regulating NF-κB signaling and Wnt5a.

## Materials and Methods

### Reagents

Lipopolysaccharides (LPS) (*Escherichia coli* O111: B4) were purchased from Sigma-Aldrich (United States). An adipogenic differentiation medium of human mesenchymal stem cells was obtained from Procell Life Science &Technology Co., Ltd., (Wuhan, China). Antibodies for flow cytometry—CD73, CD90, CD105, and CD45—were purchased from Elabscience (Wuhan, China). Antibodies for Western blot were procured from suppliers as follows: CD9, CD81, and GAPDH were acquired from Elabscience (Wuhan, China); ALIX and HSP70 were obtained from Abcam (Cambridge, United Kingdom); Wnt5a, p65, and phosphorylated-p65 were bought from Cell Signaling Technology (United States); anti-rabbit HRP-conjugated antibody was from Absin (China); PKH67 was from Fluorescence (China); and 4′,6-diamidino-2-phenylindole (DAPI) were from Solarbio (Beijing, China). Enzyme-linked immunosorbent assay (ELISA) kits for TNF-α and IL-1β were obtained from Absin (China). Enzyme-linked immunosorbent assay (ELISA) kits for IL-10 were obtained from Elabscience (China).

### Isolation and Cell Culture of GMSCs and PDLSCs

The study protocol was approved by the Ethics Committee of Qingdao University (QYFYWZLL 25971), and written informed consent was obtained from all participants. Human gingival tissues were collected and cultured as reported in our previous studies (Wang et al., 2020; Zhang Y et al., 2021). Briefly, after aseptic treatment, healthy human gingival tissue was immersed in dispase II (Sigma-Aldrich; Merck KGaA, Darmstadt, Germany) (2 mg/ml) at 4°C for 15 h and was digested with collagenase I (Sigma-Aldrich; Merck KGaA, Darmstadt, Germany) (2 mg/ml) in a 37°C water bath for 40 min. PDL tissue was scraped from the middle third part of the root surface from human third molars and premolars (in subjects 18–25 years of age) extracted for orthodontics. The PDL tissue was digested with collagenase I (Sigma-Aldrich; Merck KGaA, Darmstadt, Germany) (3 mg/ml) in a 37°C water bath for 1 h. Then, these explants were seeded in Alpha-MEM (American Hyclone) with 20% fetal bovine serum (FBS, American Hyclone) and incubated at 37°C in 5% CO2. The medium was changed every 3 days, and cells spontaneously migrated from explant tissues. All experiments were performed with GMSCs and PDLSCs subcultured on the second through fifth passages.

### Characterization of GMSCs and PDLSCs

#### Colony Forming Unit-Fibroblasts Assay

GMSCs and PDLSCs were seeded in 60-mm culture dishes at a density of 150 cells per dish with *a*-MEM with 10% FBS and cultured for 12 days. Then the cells were treated with 4% paraformaldehyde and phosphate-buffered saline (PBS) containing 1% crystal violet. Cell clusters containing >50 cells were counted as single colony-forming unit fibroblasts (CFU-F) under the microscope.

#### *In Vitro* Multipotent Differentiation

GMSCs and PDLSCs at the third passage were used for studying the capacity for differentiation into adipocytes and osteoblasts. For osteogenic differentiation, cells at 80–90% confluence were cultured in an osteogenic induction medium containing 5% FBS, 10 mM *ß*-glycerophosphate (Sigma, United States), 50 μg/ml ascorbate-2-phosphate (Sigma, United States), and 0.1 μM dexamethasone (Sigma, United States) for 28 days. For adipogenic differentiation, cells at 100% confluence were cultured in an adipogenic differentiation medium of human mesenchymal stem cells (Procell Life Science &Technology Co., Ltd., China) for 21 days.

#### Flow Cytometry

GMSCs and PDLSCs from the third through fifth passages were used for flow cytometry. Cells were collected and washed twice with PBS and then incubated with antibodies (CD45-PE, CD73-FITC, CD90-FITC, and CD105-FITC) at 4°C in the absence of light for 40 min. The suspension was analyzed by flow cytometry (Beckman Coulter, Inc., Brea, CA, United States).

### Isolation and Characterization of GMSC-Exo

When GMSCs achieved a confluence of 70–80%, the mediums were replaced by *a*-MEM containing 10% exosome-depleted FBS (SBI, United States) and then cultured for 48 h prior to supernatant harvesting. The GMSC-conditioned mediums were collected and centrifuged at 300 × g for 10 min to remove residual cells, and the supernatants were then transferred to new tubes and centrifuged at 2000 × g for 20 min. The second supernatants were centrifuged at 10,000 × g for 30 min to eliminate cell debris and other large extracellular vesicles. Clear supernatants were passed through 0.22-mm filters and transferred into the Beckman ultracentrifugation tubes. After ultracentrifugation at 100,000 × g for 70 min twice, the precipitates were resuspended with PBS and stored at −80°C.

The concentration of GMSC-Exo was quantified by the BCA Protein Assay Kit (Elabscience Biotechnology Co., Ltd., Wuhan, China). The morphology of GMSC-Exo was observed by transmission electron microscopy (TEM). The size distribution of GMSC-Exo was measured by nanoparticle tracking analysis (NTA). Exosome surface markers, including CD9, CD81, HSP70, and ALIX, were detected by Western blot (WB).

### Cellular Uptake of GMSC-Exo

GMSC-Exo labeled with PKH6 were centrifuged at 100,000 × g for 70 min and resuspended with PBS. The green fluorescence-labeled GMSC-Exo were incubated with PDLSCs for 24 h. Then, the cells were washed with PBS twice, and PDLSC nuclei were stained with 4′,6-diamidino-2-phenylindole (DAPI) for 5 min. Fluorescence signals were analyzed using a confocal microscope.

### CCK-8 Assay

Cell viability and proliferation were evaluated by the Cell Counting Kit-8 (Solarbio, China) in accordance with the manufacturer’s protocols. PDLSCs were seeded in 96-well plates (2 × 10^3^ cells/well) and subsequently treated with LPS (10 μg/ml) or GMSC-Exo (30 μg/ml). The OD values were documented from day 1 to day 4. The absorbance was measured at 450 nm.

### Coincubation of GMSC-Exo With PDLSCs

PDLSCs were cultured in 6-cell plates and treated as follows: PBS, GMSC-Exo (30 μg/ml), LPS (10 μg/ml), and LPS (10 μg/ml) + GMSC-Exo (30 μg/ml). After LPS pretreatment for 24 h, GMSC-Exo were added, cells were incubated for 24 h, and subsequent experiments were performed to detect the relevant indexes.

### Enzyme-Linked Immunosorbent Assay

The supernatants were collected after 24 h of culture and subsequently analyzed by using the corresponding ELISA kits to detect the concentrations of cytokines (TNF-α, IL-1β, and IL-10) following the manufacturer’s instructions.

### Quantitative Real-Time PCR Analysis

Total RNA from PDLSCs was extracted by TRIzol reagent (Takara, Kyoto, Japan), and cDNA was synthesized by using the Prime ScriptTM RT reagent kit (Takara, Kyoto, Japan). The mRNA levels were analyzed using SYBR Premix Ex Taq (Takara, Japan) using the 2^−Δ ΔCT^ method on the Light Cycler 480 real-time PCR system in accordance with the manufacturer’s instructions. Glyceraldehyde-3-phosphate dehydrogenase (GAPDH) was used as an internal control. The primer sequences used in this study are listed in Table 1.

**TABLE 1:** Primer sequences of qRT-PCR analysis.

Gene	Primer sequence (5–3′)
TNF-α	Forward: AGC CCT GGT ATG AGC CCA TAT ATC
	Reverse: TCC CAA AGT AGA CCT GCC CAG AC
IL-1β	Forward: TGG CTT ATT ACA GTG GCA ATG AGG ATG
	Reverse: TGT AGT GGT GGT CGG AGA TTC GTA G
IL-10	Forward: GCC AAG CCT TGT CTG AGA TGA TCC
	Reverse: GCT CCA CGG CCT TGC TCT TG
p65	Forward: CTG CCG CCT GTC CTT TCT CAT
	Reverse: ATG TCC TCT TTC TGC ACC TTG TCA C
Wnt5a	Forward: GAC TTC CGC AAG GTG GGT GAT G
	Reverse: GTC TTG TGT GGT GGG CGA GTT G
GAPDH	Forward: TGC ACC ACC AAC TGC TTA GC
	Reverse: ATG GAC TGT GGT CAT GAG

### Western Blot Analysis

Total protein was extracted from the treated PDLSCs, and the concentrations were detected and quantified by BCA assay (Elabscience, China). The same amounts of protein samples were separated by SDS-PAGE and electro-transferred onto polyvinylidene fluoride membranes. The membranes were blocked with 5% non-fat milk for 2 h and incubated with appropriate primary antibodies:rabbit anti-CD9 (1:1000), anti-CD81 (1:1000), anti-Alix (1:1000), anti-HSP70 (1:1000), anti-p65 (1:1000), anti-phosphorylated-p65 (1:1000), anti-Wnt5a (1:1000), and anti-GAPDH (1:1000) overnight at 4°C. Next, the membranes were incubated with an anti-rabbit HRP-conjugated antibody (1:5000) for 1 h at room temperature. Protein bands were visualized by the electrochemiluminescence (ECL) method.

### Statistical Analysis

Statistical analyses were performed with GraphPad Prism 8.0 (GraphPad Software, La Jolla, CA, United States). Differences between groups were determined using unpaired Student’s t-test. Data were expressed as means ± SEM, and a *p*-value <0.05 was considered statistically significant.

## Results

### Characterization of GMSCs and PDLSCs

**FIGURE 1:**
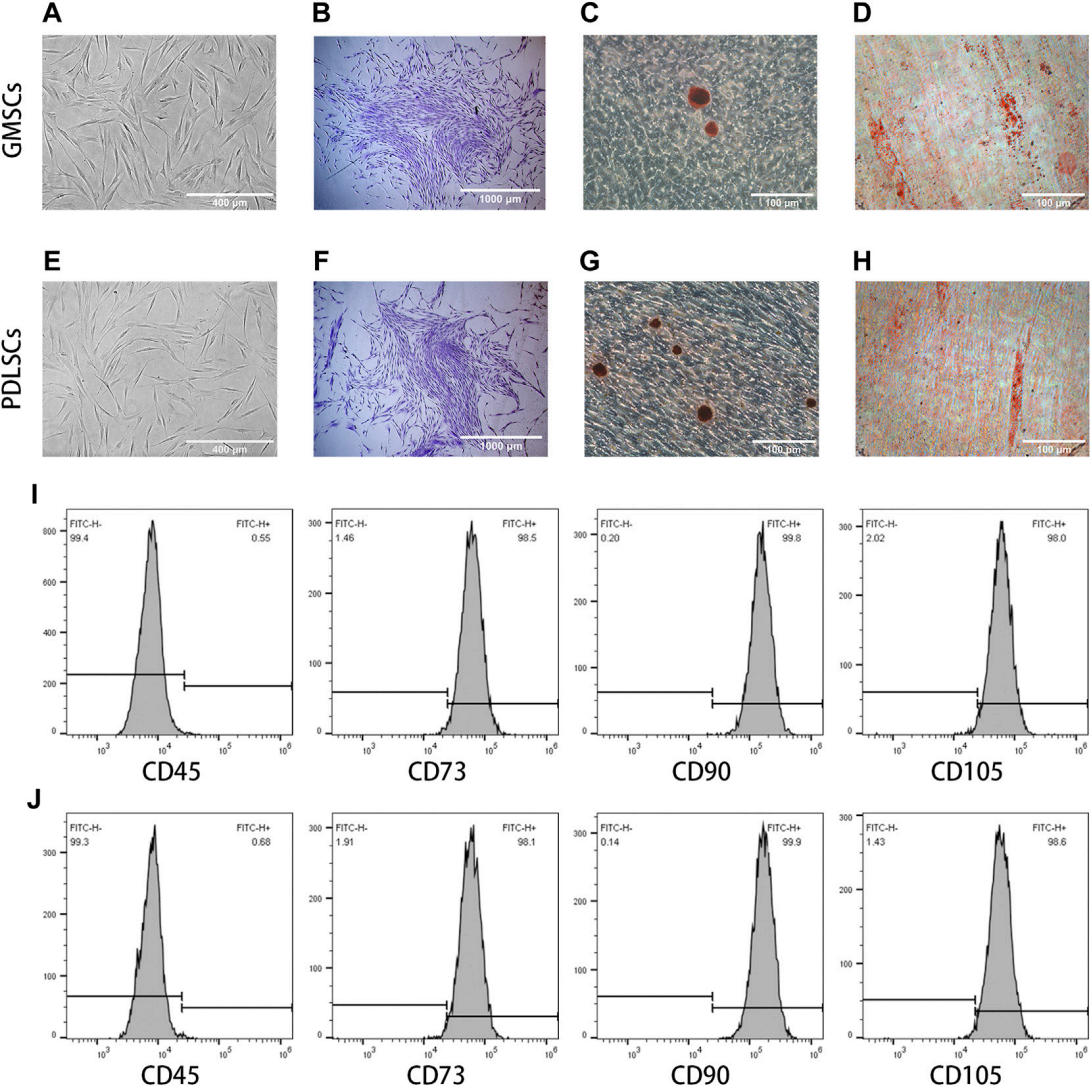
Characterization of GMSCs and PDLSCs. **(A)** Cultured GMSCs showed spindle-shaped or fibroblast-like phenotypes. **(B)** Colony-forming unit-fibroblast assay of GMSCs. **(C)** Osteogenic differentiation of GMSCs identified by Alizarin Red staining. **(D)** Adipogenic differentiation of GMSCs identified by Oil Red O staining. **(E)** Cultured PDLSCs showed spindle-shaped or fibroblast-like phenotypes. **(F)** Colony-forming unit-fibroblast assay of GMSCs. **(G)** Osteogenic differentiation of PDLSCs identified by Alizarin Red staining. **(H)** Adipogenic differentiation of PDLSCs identified by Oil Red O staining. **(I)** Flow cytometry assays revealed GMSCs expressed CD73, CD90, and CD105, but not CD45. **(J)** Flow cytometry assays revealed PDLSCs expressed CD73, CD90, and CD105, but not CD45.

Cultured GMSCs had a spindle-shaped or fibroblast-like morphology (Figure 1A) with the ability to form CFU-F *in vitro* (Figure 1B). After 28 days of osteogenic differentiation, Alizarin Red staining showed that GMSCs were able to form mineralized nodules and had potential for osteogenic differentiation (Figure 1C). After 21 days of adipogenic differentiation, Oil Red O staining displayed that GMSCs were able to form lipid droplets and had the potential to differentiate into adipocytes (Figure 1D). Flow cytometry assay revealed positive expression of the surface markers CD73, CD90, and CD105 and negative expression of CD45 (Figure 1I).

Cultured PDLSCs had a spindle-shaped or fibroblast-like morphology (Figure 1E) with the ability to form CFU-F *in vitro* (Figure 1F). Alizarin Red staining and Oil Red O staining indicated the formation of mineralized nodules and lipid droplets, suggesting the potential of PDLSCs to differentiate into osteoblasts and adipocytes (Figures 1G,H). Flow cytometry assay revealed PDLSCs expressed CD73, CD90, and CD105, but not CD45 (Figure 1J).

### Characterization of GMSC-Exo

**FIGURE 2:**
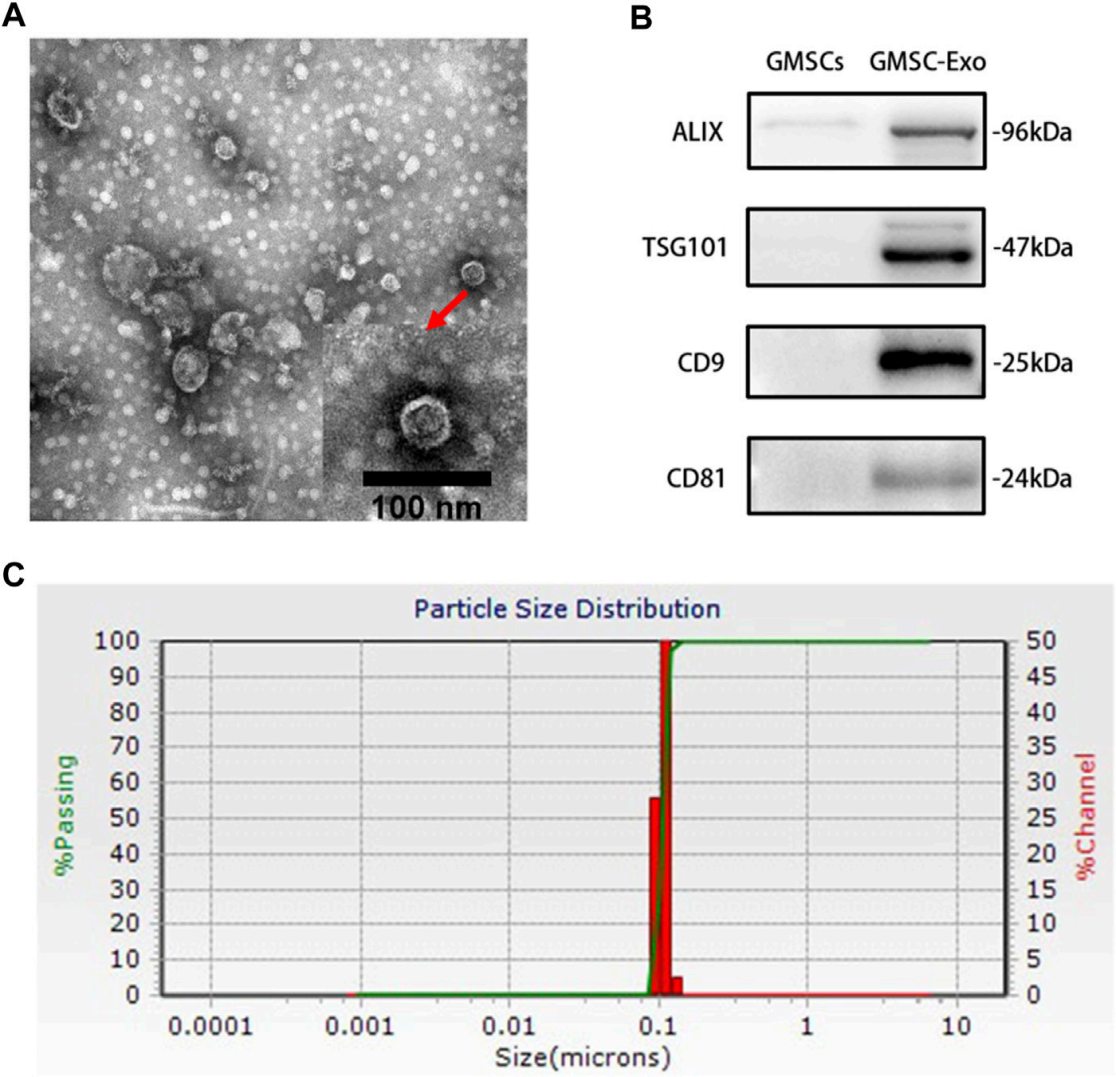
Characterization of GMSC-Exo. **(A)** GMSC-Exo were observed by transmission electron microscopy (TEM). **(B)** Western blot analysis showing the expression of ALIX, TSG101, CD9, and CD81 in GMSCs and GMSC-Exo. Equal amounts of protein (10 μg per lane) were loaded. **(C)** The particle size distribution analysis of GMSC-Exo using the nanoparticle tracking analysis (NTA), PDI = 0.0478.

TEM showed the typical cup-shaped appearance of GMSC-Exo, indicating pure exosome preparations (Figure 2A). WB confirmed that GMSC-Exo preparations were enriched with exosomal positive markers ALIX, TSG101, CD9, and CD81 (Figure 2B). NTA revealed that the majority of particles were distributed in approximately 100 nm (Figure 2C).

### Uptake of GMSC-Exo by PDLSCs

**FIGURE 3:**
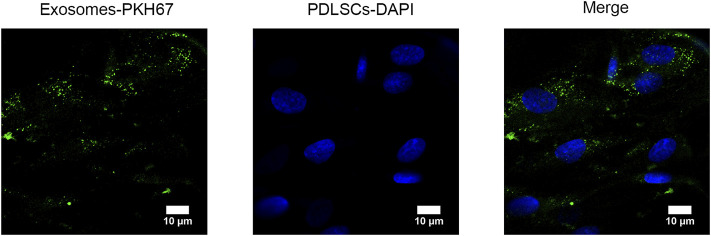
Uptake of GMSC-Exo by PDLSCs. After incubation with PDLSCs for 24 h, PKH67-labeled GMSC-Exo (green) gathered near the DAPI-labeled nucleus (blue), as visualized by the confocal microscope, indicating the uptake of GMSC-Exo by PDLSCs.

GMSC-Exo were labeled with green fluorescence by PKH67, and the nucleus of PDLSCs was labeled with blue fluorescence by DAPI. After incubation with PDLSCs for 24 h, the green fluorescence-labeled GMSC-Exo gathered near the blue fluorescence-labeled nucleus, as visualized by using the confocal microscope, indicating the uptake of GMSC-Exo by PDLSCs (Figure 3).

### Effect of GMSC-Exo on the Proliferation of PDLSCs

**FIGURE 4:**
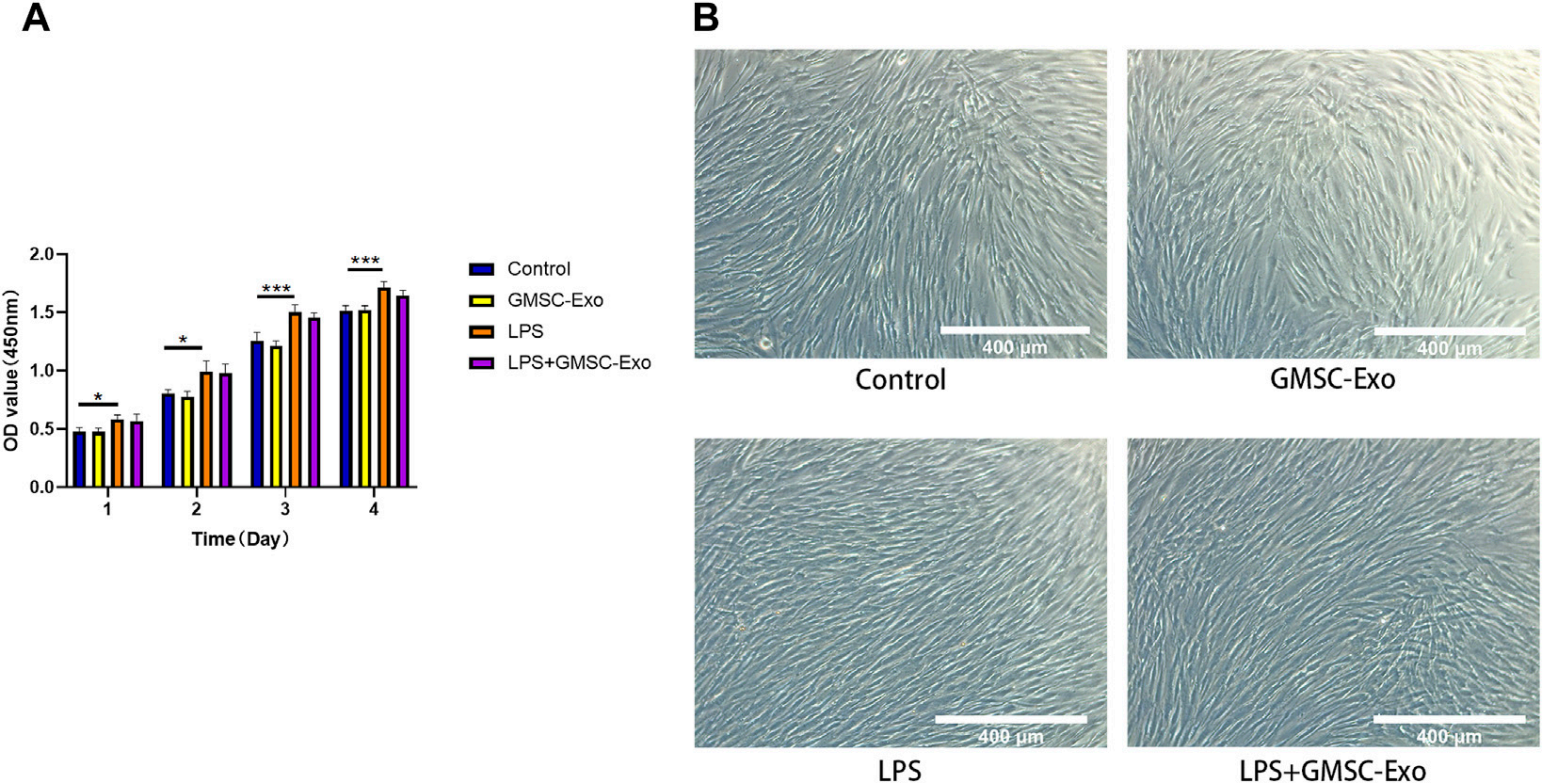
Effect of GMSC-Exo on the proliferation of PDLSCs. **(A)** The cell proliferation curve of PDLSCs from day 1 to day 4 (*p < 0.05, **p < 0.01, ***p < 0.001). **(B)** Changes in cell morphology of PDLSCs after treatment with GMSC-Exo or LPS.

CCK-8 assays were used to detect the effects of GMSC-Exo on PDLSC proliferation in the LPS-induced inflammatory microenvironment. Compared to the control group, treatment with GMSC-Exo showed no substantial effect on the proliferation of PDLSCs (Figure 4A). Treatment with LPS (10 μg/ml) significantly promoted the proliferation of PDLSCs (Figures 4A,B). Compared with the control group, the LPS-treated cells changed from long spindle-shaped or fibroblast-like morphology to a short spindle-shaped morphology (Figure 4B). Co-treatment with LPS and GMSC-Exo had no significant effect on the proliferation capacity of PDLSCs compared to treatment with LPS on the first and second days. Although co-treatment with GMSC-Exo and LPS decreased the cell proliferation capacity on the third and fourth days compared with LPS treatment, it was not statistically significant (Figure 4A).

### GMSC-Exo Reduce the Inflammatory Response of PDLSCs Induced by LPS

**FIGURE 5:**
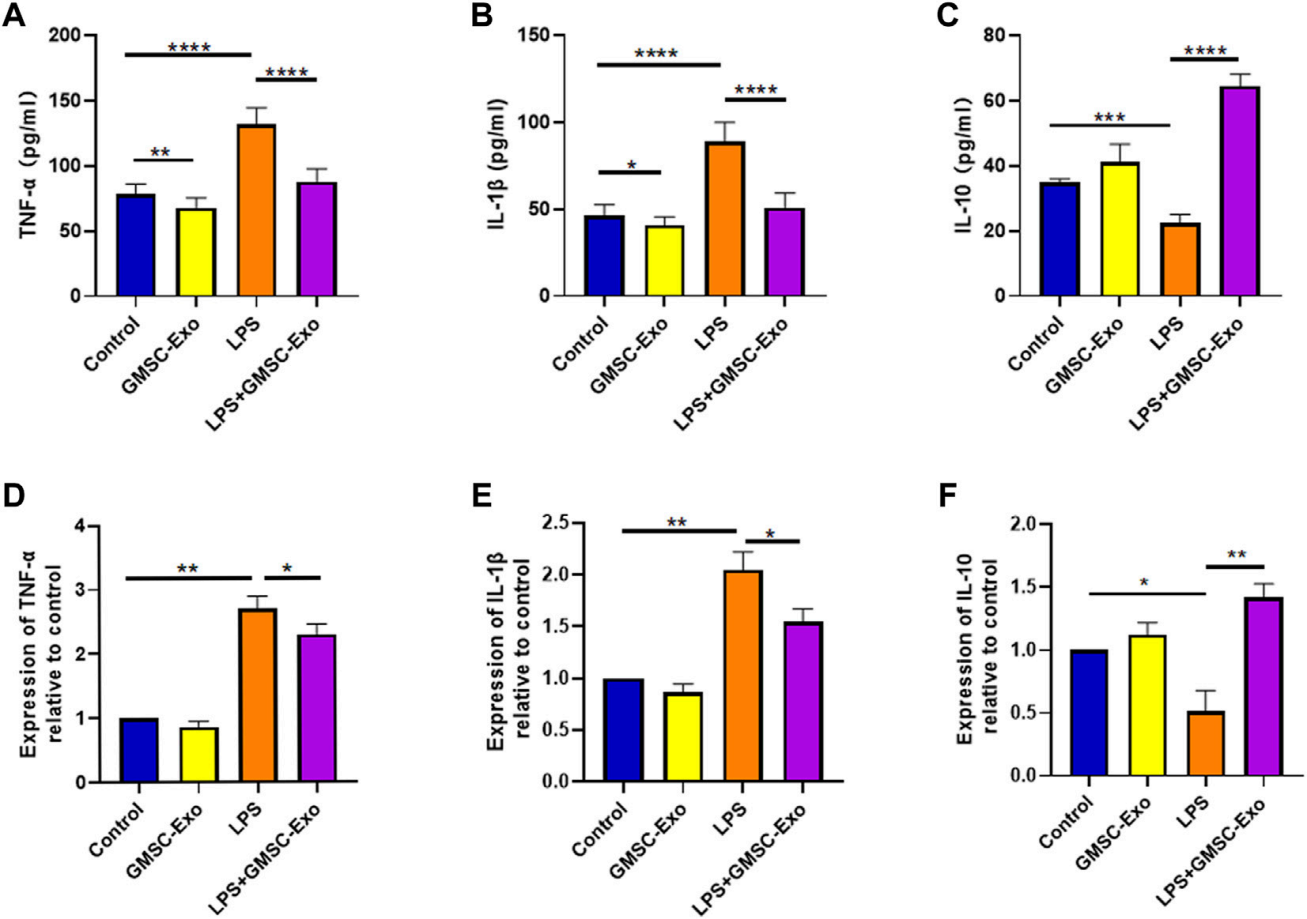
GMSC-Exo reduces the upregulation of pro-inflammatory factors in PDLSCs induced by LPS. **(A–C)** The expression of TNF-α, IL-1β, and IL-10 in supernatants was measured by ELISA. **(D–F)** Expression of TNF-α, IL-1β, and IL-10 mRNAs was analyzed by qRT-PCR (*p < 0.05, **p < 0.01, ****p < 0.0001).

To investigate whether GMSC-Exo could influence the inflammatory response of LPS-induced PDLSCs, the expression of TNF-α, IL-1β, and IL-10 was measured by ELISA and qRT-PCR. Compared with the control group, treatment with LPS significantly increased the levels of TNF-α in supernatants and decreased the levels of the anti-inflammatory factor IL-1β. This abnormal change was reversed by the GMSC-Exo treatment (Figures 5A–C). The qRT-PCR results also showed that the overexpression of TNF-α and IL-1β mRNAs in LPS-induced PDLSCs was inhibited by GMSC-Exo (Figures 5D,E). IL-10, which is downregulated by LPS treatment, was upregulated by GMSC-Exo (Figure 5F). These results suggest that GMSC-Exo can attenuate the inflammatory response in PDLSCs induced by LPS.

### GMSC-Exo Affect NF-κB Signaling and Wnt5a in Reducing the Inflammatory Response in PDLSCs Induced by LPS

**FIGURE 6:**
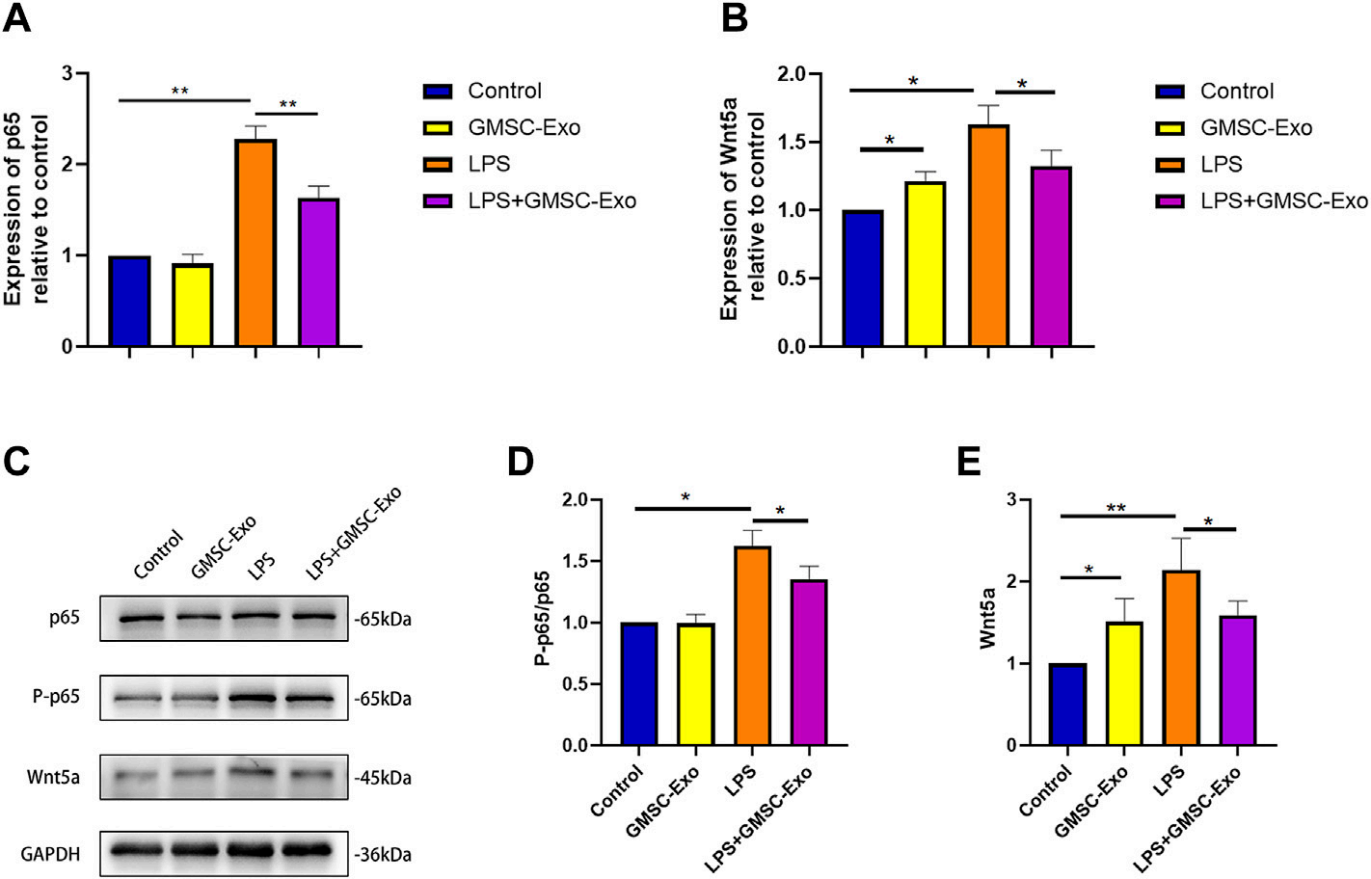
GMSC-Exo affect NF-κB signaling and Wnt5a in reducing the inflammatory response in PDLSCs induced by LPS. **(A,B)** Gene expression of p65 and Wnt5a was analyzed by qRT-PCR. **(C)** Western blot analysis showed the protein expression of p65, P-p65, and GAPDH was used as the internal control. **(D,E)** Relative intensity of the tested protein (*p < 0.05, **p < 0.01).

It has been clarified that the NF-κB signaling pathway and Wnt5a are involved in inflammation. To ascertain the mechanism by which GMSC-Exo inhibit the inflammatory response in PDLSCs, we investigated the expression of NF-κB and Wnt5a after treatment with GMSC-Exo. As shown in Figures 6A,B, qRT-PCR results indicated that LPS upregulated the expression of the NF-κB pathway (p65) and Wnt5a, and co-treatment with GMSC-Exo significantly suppressed this overexpression. Western blot showed similar results (Figures 6C–E). These results suggest that GMSC-EXO attenuate LPS-induced inflammation by inhibiting the NF-κB pathway and Wnt5a. However, treatment with GMSC-Exo without LPS enhanced the expression of Wnt5a.

## Discussion

Periodontitis results from complex interactions between the microflora and the dysregulated host’s immuno-inflammatory response (Grossi et al., 1994). PDLSCs are important for periodontal homeostasis and regeneration (Tomokiyo, Wada, and Maeda, 2019). However, the regenerative capacity of PDLSCs in patients with periodontitis is significantly reduced because of periodontal tissue inflammation. The inhibition of inflammatory responses may represent a promising approach to restore the regenerative capacity of PDLSCs and generate cementum/PDL-like tissue and alveolar bone destroyed by periodontitis. Studies have confirmed the anti-inflammatory and immunosuppressive effects of MSC-derived exosomes (Pivoraitė et al., 2015; Ha et al., 2020). In addition, exosomes not only have similar therapeutic potential in their secretory cells but can also overcome numerous disadvantages of cellular therapy (Jafarinia et al., 2020). In view of this, MSC-Exo therapy appears to be a novel therapy for inflammatory diseases. Compared with other MSCs, GMSCs are thought to be the best stem cell source for cellular therapies and regenerative treatments because of the availability and accessibility of gingival tissues, their unique immunomodulatory functions, and excellent self-renewal and multipotent differentiation properties (Grawish, 2018). Moreover, our previous work has demonstrated that GMSC-Exo play an important role in the regulation of immuno-inflammatory responses (Wang et al., 2020; Zhang Y et al., 2021). Therefore, we investigated whether and how GMSC-Exo could modulate PDLSCs in the inflammatory microenvironment.

In the present study, PDLSCs and GMSCs from periodontal and gingival tissues were separated and characterized. GMSC-Exo were isolated from the supernatants of GMSC cultures *via* ultracentrifugation and identified by TEM, NTA, and Western blot. We observed the uptake of GMSC-Exo by PDLSCs after co-incubation for 24 h. The proliferation of PDLSCs is promoted by the inflammatory microenvironment (Li et al., 2014). Treatment with LPS enhances the proliferation of PDLSCs (Liu et al., 2019), which is consistent with our results. In addition, our results showed that treatment with GMSC-Exo had no significant influence on the abnormal proliferation of PDLSCs caused by LPS.

TNF-α and IL-1β are critical inflammatory mediators that play an important role in the interactions between PDLSCs and immune cells (Behm et al., 2020), which are significant in the development of periodontitis (Pan, Wang, and Chen, 2019). Available evidence suggests that TNF-α and IL-1β are vital in the osteogenesis of PDLSCs in the inflammatory environment (Mao et al., 2016; Chen et al., 2020). A previous study has reported that GMSC-Exo could inhibit the secretion and expression of pro-inflammatory cytokines, including TNF-α and IL-1β, in LPS-induced inflammatory macrophages (Wang et al., 2020). IL-10, an anti-inflammatory factor, was revealed to dampen IL-17-mediated periodontitis-associated inflammatory network (Sun et al., 2020). It has been shown that elevated levels of IL-10 help promote periodontal tissue regeneration (Liu H et al., 2019). In this study, ELISA revealed that GMSC-Exo treatment reduced the secretion of TNF-α and IL-1β induced by LPS, and qRT-PCR showed the decreased mRNA levels of TNF-α and IL-1β. In addition, IL-10, which was lowly expressed induced by LPS, was upregulated by GMSC-Exo treatment. The above results indicate that GMSC-Exo have an anti-inflammatory effect on PDLSCs in the LPS-induced inflammatory microenvironment. We then investigated the mechanism by which GMSC-Exo produced this effect. Interestingly, it is reported that TNF-α preconditioned GMSC-Exo showed a more significant anti-inflammatory effect than GMSC-Exo (Nakao et al., 2021). It suggests that we need to further investigate the therapeutic potential of LPS-preconditioned GMSC-Exo.

As an important nuclear transcription factor, the NF-κB signaling pathway has shown to be involved in inflammation, immune response, and cell proliferation and differentiation. NF-κB signaling regulates inflammation by controlling the expression of pro- and anti-inflammatory genes (Taniguchi and Karin, 2018). It has been shown that inhibiting the NF-κB pathway can attenuate the impaired osteogenesis in PDLSCs under TNF-α-induced inflammatory conditions (Zhang W et al., 2021). MSC-Exo have been shown to attenuate inflammatory responses *via* suppressing the NF-κB pathway in multiple disease models (Xian et al., 2019; Zhang W et al., 2019; Williams et al., 2020). In this study, NF-κB signaling (p65) was activated in PDLSCs in the LPS-induced inflammatory microenvironment, and the activation event was reversed by GMSC-Exo. The result was in line with the changes in TNF-α and IL-1β. In conclusion, these data suggest that GMSC-Exo have an inhibitory effect on the NF-κB pathway in LPS-induced inflammatory PDLSCs. The NF-κB signaling pathway and a Wnt5a-related non-canonical Wnt signaling pathway are closely linked to inflammation. Recent findings suggest that there is complex cross-talk between the two signaling pathways (Lopez-Bergami and Barbero, 2020). We therefore propose that GMSC-Exo suppressed NF-κB signaling related to Wnt5a.

Wnt5a has been acknowledged to participate in the pathogenesis of periodontitis, take part in the development of periodontium, promote fibrinogenesis, and moderate osteogenesis in periodontal stem cells (Wei et al., 2021). In early periodontitis, Wnt5a was overexpressed in periodontal ligament tissue and regulated the CaMKII pathway, which should be involved in maintaining periodontal homeostasis (Qian et al., 2021). It has been demonstrated that Wnt5a modulated the expression of receptor activation of the NF-κB ligand (RANKL) (Nakao et al., 2021). Moreover, GMSC-Exo were shown to regulate the RANKL/osteoprotegrin (OPG) ratio by targeting Wnt5a-mediated RANKL expression in PDLSCs and inhibiting its osteoclastogenic ability (Nakao et al., 2021). Wnt5a is usually at a low expression level in healthy adult tissues compared to those of the embryo (Yap et al., 2014), but the dysregulation of Wnt5a usually results in it being overexpressed, resulting in a number of diseases. It was significantly elevated in gingival crevicular fluid at periodontitis sites when compared to healthy gums (Chatzopoulos et al., 2021). The expression of Wnt5a was upregulated in the inflammatory microenvironment simulated by LPS *in vitro* (He et al., 2014), which was in agreement with our results. Here, we demonstrated that GMSC-Exo-attenuated LPS-induced Wnt5a activity in PDLSCs is consistent with the expression of TNF-α and IL-1β. These results indicate that the upregulation of Wnt5a in LPS-induced PDLSCs can be suppressed by GMSC-Exo. Interestingly, compared to the control, qRT-PCR and Western blot showed that treatment with GMSC-Exo upregulated the expression of Wnt5a. However, the levels of inflammatory factors did not show a significant increase. We speculate that the overexpression of Wnt5a may be related to osteogenesis, rather than inflammation. It was reported that Wnt5a inhibited osteogenic differentiation of PDLSCs through ROR2/JNK signaling (Hasegawa et al., 2018). However, combination of Wnt5a with 100 ng/ml BMP2 significantly promoted the osteogenic differentiation of dental follicle stem cells (Xiang et al., 2014). These pieces of evidence suggest that Wnt5a has a bidirectional function on the osteogenic differentiation of periodontal stem cells (Wei et al., 2021). We will further investigate the significance of GMSC-Exo regulation of Wnt5a in the osteogenesis and periodontal tissue regeneration of PDLSCs in future studies.

We demonstrated that GMSC-Exo attenuated LPS-induced inflammatory responses by suppressing the expression of NF-κB and Wnt5a in PDLSCs. Emerging evidence has shown that LPS derived from *E. coli* affects the osteogenesis of PDLSCs and human dental pulp stem cells through the toll-like receptor 4 (TLR4)-mediated NF-κB pathway and Wnt5a (Li et al., 2014; He et al., 2014; P.; Wang et al., 2021). Čebatariūnienė et al. (2019) determined that exosomes from PDLSCs could suppress NF-κB signaling, while combined treatment with exosomes and anti-TLR4 blocking antibodies attenuated the inhibitory effect on the NF-κB activity, indicating that the effect might be achieved *via* competition for TLR4. The idea that the TLR4/NF-κB signaling pathway is a potential target of exosomes is also supported by other studies (W. Li W et al., 2021; J. Liu et al., 2019b; Q. Li Q et al., 2021). Wnt5a was overexpressed in human macrophages infected with *Porphyromonas gingivalis*, which could be downregulated by TLR4 neutralizing antibody or inhibitor. It is proposed that Wnt5a is linked to TLR4-induced host inflammatory response in peri-implantitis (Zhang et al., 2020). In addition, LPS enhanced the expression of Wnt5a through TLR4 in human dental pulp stem cells (He et al., 2014), and TLR4 knockdown decreased the expression of Wnt5a, which was related to osteogenesis and chondrogenesis in MSCs derived from human bone marrow (Khodabandehloo et al., 2021). We therefore speculated that GMSC-Exo might regulate the inflammatory response through competition for TLR4 to regulate NF-κB signaling and Wnt5a. However, the hypothesis requires further research and demonstration.

Studies have confirmed the intricate relationship between NF-κB and Wnt5a (Lopez-Bergami and Barbero, 2020). He and coworkers (2014) demonstrated that LPS enhanced Wnt5a expression through the TLR4/MyD88/NF-κB pathway and PI3K/AKT/NF-κB pathway in human dental pulp stem cells and that the NF-κB pathway inhibitor significantly inhibited the overexpression. Also, it has been found that upregulation of mesenchymal expression of Wnt5a induced by hyperoxic injury in the developing lung was suppressed by a specific inhibitor of NF-κB signaling, BAY11–7082, which inhibits phosphorylation of IκBα (nuclear factor of kappa light polypeptide gene enhancer in B-cell inhibitor, α) (Sucre et al., 2020). The aforementioned findings suggest that NF-κB regulates Wnt5a. However, more studies indicate that Wnt5a is upstream of NF-κB and regulates NF-κB expression through a complex set of signaling pathways. ROR1 and FZD5 are receptors for Wnt5a, which have been shown to be upstream in the activation of NF-κB by Wnt5a (Barbero et al., 2019; Chen et al., 2019; Tai et al., 2021). Moreover, LPS has been reported to activate the Wnt5a/JNK/NF-κB pathway in H9c2 cells (Yu et al., 2020). Pretreatment with the JNK inhibitor SP60025 significantly reduced the levels of NF-κB in the nucleus which were higher than those induced by silenced Wnt5a (siWnt5a), indicating that other signaling pathways are involved in the Wnt5a/NF-κB pathway besides JNK (Yu et al., 2020). The same results were obtained in LPS-induced fibroblast-like synoviocytes (Xie et al., 2020). Zhao et al. (2015) proposed that the upregulation of Wnt5a mediated a positive feedback loop in the inflammatory response in polycystic ovary syndrome. LPS could activate NF-κB signaling *via* a toll-like receptor, inducing the expression of Wnt5a which was related to inflammatory factors. Wnt5a, in turn, can further stimulate the production of inflammatory factors and chemokines through the PI3K/AKT/NF-κB signaling pathway, forming a positive feedback loop that continuously amplifies the Wnt5a-mediated inflammatory response (Zhao et al., 2015). In addition, a negative feedback loop between Wnt5a and NF-κB in inflammation-driven intervertebral disc degeneration has been described. The expression of Wnt5a was induced by TNF-α/NF-κB signaling, and on the other hand, Wnt5a suppressed TNF-α-induced activation of NF-κB signaling and downstream inflammatory gene expression in return (Li et al., 2018). However, in the present study, it is difficult to confirm which one of NF-κB or Wnt5a was upstream. We will further delve into the specific mechanisms of cross-talk between NF-κB signaling and Wnt5a in the regulation of inflammatory response by GMSC-Exo.

## Conclusion

Our present study reveals that GMSC-Exo exert the therapeutic effect by regulating NF-κB signaling and Wnt5a. GMSC-Exo can inhibit the production of inflammatory factors and promote the production of anti-inflammatory factors in LPS-induced PDLSCs. Attenuating the inflammatory response of PDLSCs helps restore their regenerative potential, which is impaired by inflammation. NF-κB signaling and Wnt5a were essential for the suppression of inflammation *via* GMSC-Exo. In addition, the upregulation of Wnt5a induced by GMSC-Exo may be related to the osteogenesis of PDLSCs, rather than inflammation. In conclusion, GMSC-Exo may be a novel therapeutic approach against periodontitis and other inflammatory disorders.

## Data Availability

The original contributions presented in the study are included in the article/Supplementary material; further inquiries can be directed to the corresponding author.
